# vvECMO can be avoided by a transpulmonary pressure guided open lung concept in patients with severe ARDS

**DOI:** 10.1186/s13054-019-2421-x

**Published:** 2019-04-23

**Authors:** Philip van der Zee, Dinis Dos Reis Miranda, Han Meeder, Henrik Endeman, Diederik Gommers

**Affiliations:** Department of Adult Intensive Care, Erasmus MC, Doctor Molewaterplein 40, 3015 GD Rotterdam, the Netherlands

Dear Editor,

The EOLIA trial concluded that vvECMO compared to conventional mechanical ventilation with low tidal volumes and airway pressures ≤30 cmH_2_O did not improve survival [1]. Although not statistically significant, the 11% absolute reduction in mortality rate and multiple crossovers to rescue vvECMO were considered to be clinically relevant [2]. However, a conventional mechanical ventilation strategy is likely to be insufficient for patients with severe ARDS, as higher airway pressures are required to maintain lung aeration [3]. Grasso et al. measured the transpulmonary pressure (P_L_) in patients with severe ARDS and increased PEEP until P_L_ was 25 cmH_2_O. Fifty percent of patients responded to an increase in airway pressure and did not require vvECMO [4]. We hypothesized that a P_L_ guided open lung concept (OLC) could improve oxygenation and prevent conversion to vvECMO in patients with severe ARDS.

We retrospectively reviewed the records of all patients referred to our ICU between January and May 2018. Eight patients had severe ARDS and had an indication for vvECMO according to the EOLIA trial (demographics are given in the Additional file 1) [1]. Before referral protective mechanical ventilation with low tidal volume and a plateau pressure of approximately 30 cmH_2_O was applied. PaO_2_/FiO_2_ ratio was 62 ± 7 mmHg despite the use of neuromuscular blocking agents and prone positioning. After referral, a recruitment maneuver was performed and PEEP was increased. P_L_ was estimated with an esophageal balloon catheter and we aimed for a P_L_ ≤ 25 cmH_2_O. In addition, respiratory rate and I:E ratio were increased, thereby generating intrinsic PEEP.

The P_L_ guided OLC resulted in an increase in PaO_2_/FiO_2_ ratio to 201 ± 87 mmHg (Fig. 1) and none of the patients required vvECMO. During the first 6 h peak airway pressure was increased to 44.9 ± 10.2 cmH_2_O, but was reduced to 36.3 ± 5.6 cmH_2_O within 24 h, while PEEP was maintained at 20.6 ± 4.0 cmH_2_O. A maximum end-inspiratory P_L_ of 18 ± 5 cmH_2_O was measured. At 72 h both peak airway pressures and PEEP were reduced to baseline values while oxygenation remained stable.

**Fig. 1:**
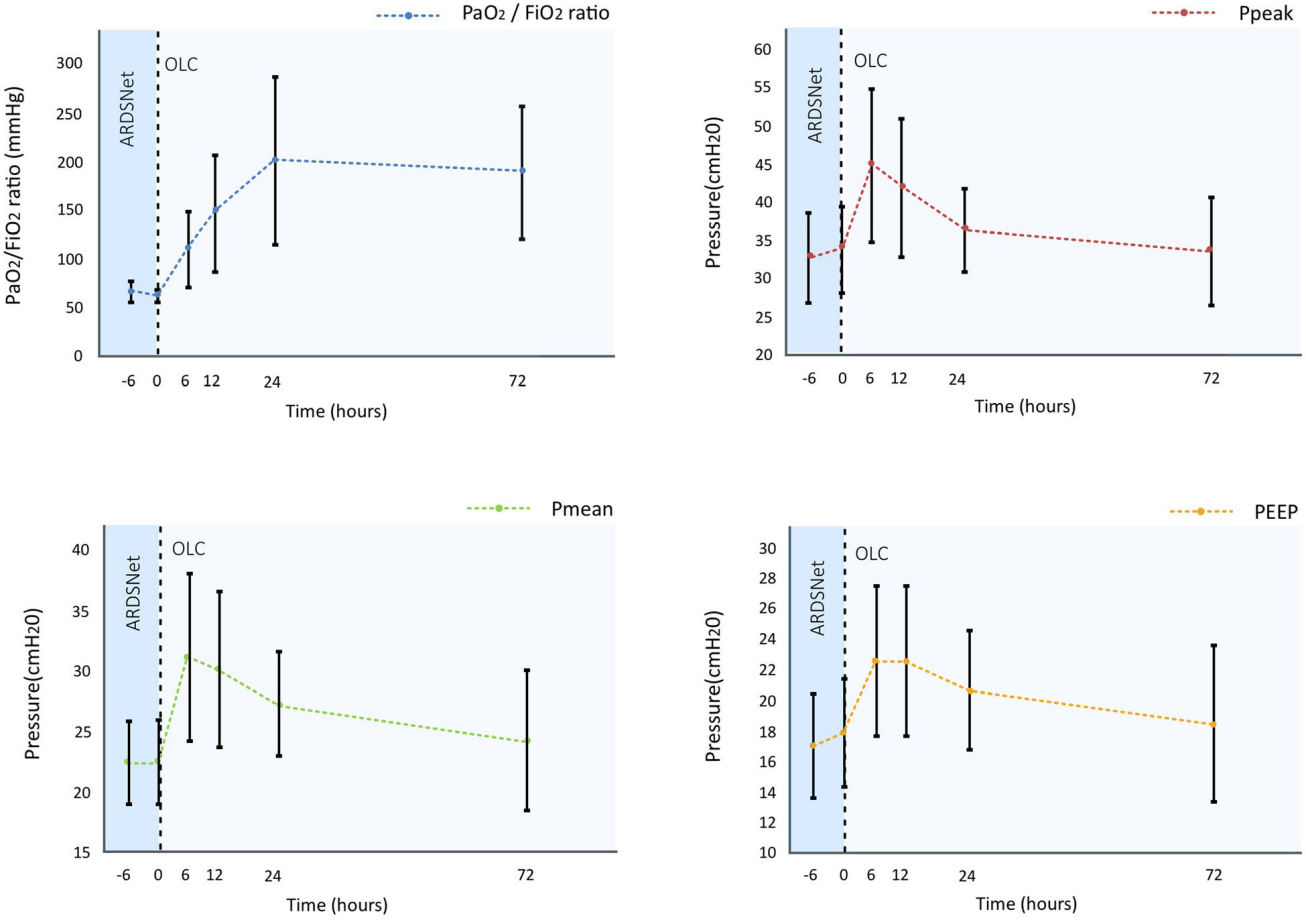
Airway pressures and PaO_2_ / FiO_2_ ratio after initiation of the OLC. Peak airway pressure, Pmean, PEEP and PaO_2_ / FiO_2_ ratio as a function of time. The OLC is initiated at T0, i.e. at referral. Mean values and standard deviations are shown. Note that PEEP values are set PEEP levels at the mechanical ventilator. The depicted driving pressure is overestimated as intrinsic PEEP is not shown. FiO_2_ fraction of inspired oxygen, PaO_2_ partial pressure of arterial oxygen, Ppeak peak airway pressure, Pmean mean airway pressure, PEEP positive end-expiratory pressure

These data suggest that the OLC improves oxygenation and avoids conversion to vvECMO in patients with severe ARDS. We acknowledge that a recruitment maneuver and higher PEEP in patients with moderate to severe ARDS increased mortality in the Alveolar Recruitment Trial [5]. However, the recruitment maneuver was standardized and ‘recruitability’ was not assessed. We hypothesize that a recruitment maneuver and higher PEEP is beneficial in patients with large regions of decreased lung aeration. Thus, future research should focus on individual ‘recruitability’ [6]. Baedorf Kassis et al. introduced a recruitment maneuver based on P_L_ measurements [7]. Other potential predictors are a decrease in driving pressure, oxygenation response to PEEP-trials, or lung aeration estimated by electrical impedance tomography or ultrasound.

## Additional file

Additional file 1: **Figure S1.** Flowchart of patient inclusion. **Table S1.** Patient demographics. **Table S2.** Patient parameters. Appendix Mechanical ventilation strategy. (DOCX 38 kb)
