# Supplementary material for: vvECMO can be avoided by a transpulmonary pressure guided open lung concept in patients with severe ARDS
Source: Crit Care. 2019 Apr 23;23:133. doi: 10.1186/s13054-019-2421-x (PMC6480621; doi:10.1186/s13054-019-2421-x)
Supplement: Supplementary file 1 — Figure S1. Flowchart of patient inclusion. Table S1. Patient demographics. Table S2. Patient parameters. Appendix Mechanical ventilation strategy. (DOCX 38 kb) [file 13054_2019_2421_MOESM1_ESM.docx]

**Supplementary files**

**Figure S1. Flowchart of patient inclusion**

**61** ICU referrals between January and May 2018

**50** Patients did not meet Berlin definition of ARDS

**11** Patients met Berlin definition of ARDS[7]

**3** ARDS patients excluded

Venovenous ECMO at referring ICU (2)

Pregnancy (1)

**8** ARDS patients included

**Table S1. Patient demographics**

| **Case** | **Age** | **Gender** | **Admission Diagnosis** | **PaO_2_ / FiO_2_ ratio at referral (mmHg)** | **APACHE IV Score at referral** | **ICU LoS prior to referral (days)** | **ICU LoS (days)*** | **BMI** | **ICU survival** |
| --- | --- | --- | --- | --- | --- | --- | --- | --- | --- |
| 1 | 74 | M | Influenza, Streptococcus pyogenes | 54 | 108 | 4 | 18 | 24.2 | Survivor |
| 2 | 67 | M | Influenza | 66 | 84 | 1 | 18 | 35.8 | Survivor |
| 3 | 27 | M | Influenza | 73 | 103 | 1 | 33 | 27.8 | Survivor |
| 4 | 51 | M | Invasive aspergillosis | 69 | 98 | 5 | 9 | 20.4 | Non-survivor |
| 5 | 60 | F | Influenza | 58 | 128 | 2 | 42 | 36.7 | Survivor |
| 6 | 46 | F | Pancreatitis | 61 | 100 | 2 | 15 | 44.1 | Survivor |
| 7 | 64 | F | Influenza, Streptococcus pneumoniae | 52 | 147 | 5 | 17 | 29.4 | Survivor |
| 8 | 49 | M | Influenza | 78 | 52 | 2 | 20 | 25.5 | Survivor |

* Total length of stay at the referring ICU and the ICU of the Erasmus MC. ICU intensive care unit, LoS length of stay, BMI body mass index, M male, F female.

**Table S2. Patient parameters**

|  | | **Before referral** | **After referral** | | | | | | | |
| --- | --- | --- | --- | --- | --- | --- | --- | --- | --- | --- |
|  | | **0h (T0)** | **6h (T6)** | p-value | **12h (T12)** | p-value | **24h (T24)** | p-value | **72h (T72)** | p-value |
| **Mechanical ventilation settings** | | |  |  |  |  |  |  |  |  |
| RR (1/min) |  | 25.8 ± 3.6 | 38.4 ± 9.2 | 0.003 | 37.4 ± 8.0 | 0.002 | 37.8 ± 8.5 | 0.002 | 36.3 ± 8.3 | 0.007 |
| PEEP (cmH_2_O) | | 17.8 ± 3.5 | 22.6 ± 4.9 | 0.012 | 22.6 ± 5.0 | 0.018 | 20.6 ± 4.0 | 0.014 | 18.4 ± 5.1 | 0.738 |
| Pmean (cmH_2_O) | | 22.5 ± 3.5 | 31.1 ± 7.0^‡^ | 0.042 | 30.3 ± 6.5^‡^ | 0.105 | 27.4 ± 4.3 | 0.242 | 24.4 ± 5.8 | 0.734 |
| Ppeak (cmH_2_O) | | 33.9 ± 5.7 | 44.9 ± 10.2^†‡^ | 0.051 | 41.9 ± 8.9^†‡^ | 0.099 | 36.3 ± 5.6 | 0.437 | 33.5 ± 7.1 | 0.917 |
| I:E ratio | | 0.79 ± 0.20 | 1.51 ± 0.45^‡^ | 0.006 | 1.44 ± 0.51 | 0.025 | 1.46 ± 0.51 | 0.018 | 1.07 ± 0.43 | 0.133 |
| Vt (mL) | | 462 ± 69 | 416 ± 67 | 0.156 | 443 ± 103 | 0.611 | 428 ± 75 | 0.369 | 395 ± 56 | 0.009 |
| Vt/kg PBW | | 6.7 ± 0.6 | 6.1 ± 1.2 | 0.169 | 6.5 ± 1.7 | 0.702 | 6.3 ± 1.7 | 0.528 | 5.8 ± 0.8 | 0.006 |
| FiO_2_ (%) | | 96 ± 6 | 79 ± 17^†‡^ | 0.034 | 66 ± 19 | 0.006 | 57 ± 14 | 0.001 | 54 ± 22 | 0.003 |
| MV (L/min) | | 10.9 ± 1.0 | 16.1 ± 4.5 | 0.007 | 16.0 ± 3.6 | 0.007 | 15.3 ± 3.1 | 0.007 | 14.8 ± 2.9 | 0.007 |
| **Gas exchange** | |  |  |  |  |  |  |  |  |  |
| pH | | 7.26 ± 0.10 | 7.30 ± 0.06 | 0.116 | 7.32 ± 0.05 | 0.102 | 7.34 ± 0.04 | 0.037 | 7.36 ± 0.08 | 0.068 |
| PaCO_2_ (mmHg) | | 59.0 ± 16.2 | 50.1 ± 10.1 | 0.046 | 49.4 ± 10.7 | 0.100 | 48.3 ± 8.9 | 0.067 | 55.1 ± 15.2 | 0.403 |
| PaO_2_ (mmHg) | | 60.0 ± 7.8 | 85.6 ± 34.6 | 0.076 | 90.1 ± 19.2 | 0.001 | 104.6 ± 24.4 | 0.001 | 91.2 ± 22.7 | 0.003 |
| Bicarbonate (mmol/L) | | 25.2 ± 6.2 | 24.8 ± 6.1^‡^ | 0.404 | 25.1 ± 4.9^‡^ | 0.977 | 26.0 ± 4.6^‡^ | 0.619 | 30.2 ± 3.8 | 0.023 |
| Base excess | | -1.5 ± 8.7 | -2.3 ± 6.0^‡^ | 0.468 | -1.5 ± 4.4^‡^ | 0.982 | -0.3 ± 4.3^‡^ | 0.601 | 3.9 ± 3.2 | 0.089 |
| Oxygen saturation (%) | | 88 ± 6 | 94 ± 3^†^ | 0.028 | 97 ± 2 | 0.003 | 97 ± 2 | 0.001 | 96 ± 4 | 0.022 |
| PaO_2_ / FiO_2_ ratio | | 62 ± 7 | 110 ± 39*^†‡^ | 0.013 | 149 ± 60 | 0.005 | 201 ± 87 | 0.003 | 191 ± 68 | 0.001 |
| **Hemodynamic parameters** | | |  |  |  |  |  |  |  |  |
| Heart rate (1/min) | | 113 ± 24 | 107 ± 26 | 0.348 | 105 ± 20 | 0.113 | 104 ± 20 | 0.344 | 108 ± 16 | 0.609 |
| MAP (mmHg) | | 75 ± 12 | 73 ± 11 | 0.748 | 77 ± 8 | 0.749 | 76 ± 10 | 0.838 | 76 ± 13 | 0.875 |
| Lactate (mmol/L) | | 2.3 ± 1.0 | 2.5 ± 0.7* | 0.380 | 1.9 ± 0.5 | 0.652 | 2.0 ± 0.8 | 0.784 | 2.1 ± 1.4 | 0.912 |
| Noradrenalin (µg/kg/min) | | 0.25 ± 0.18 | 0.43 ± 0.37^‡^ | 0.110 | 0.36 ± 0.36 | 0.266 | 0.28 ± 0.23 | 0.708 | 0.26 ± 0.44 | 0.975 |

Values are mean ± standard deviation. Presented p-values are compared to T0. * p-value < 0.05 as compared to T12; † p-value < 0.05 as compared to T24; ‡ p-value < 0.05 as compared to T72. RR respiratory rate; PEEP positive end-expiratory pressure; Pmean mean airway pressure; Ppeak peak airway pressure; I:E ratio inspiratory : expiratory ratio; Vt tidal volume; PBW predicted body weight; FiO_2_ fraction of inspired oxygen; MV minute volume; PaCO_2_ partial pressure of arterial carbon dioxide; PaO_2_ partial pressure of arterial oxygen; MAP mean arterial pressure.

**Appendix Mechanical ventilation strategy**

Controlled mechanical ventilation with a strive tidal volume of 4-6ml/kg predicted body weight was applied in all patients. A recruitment maneuver was performed using stepwise increments in PEEP (usually 5 cmH_2_O) while driving pressure was maintained. Peak airway pressure was allowed approximately 20 cmH_2_O above the initial peak airway pressure for 2 minutes. The recruitment maneuver was discontinued if mean arterial pressure decreased below a predefined cut-off set by the clinician (usually <50 mmHg). Subsequently, peak inspiratory pressures were guided by transpulmonary pressures (P_L_) instead of plateau pressures. P_L_ was estimated with an esophageal balloon catheter. An inspiratory P_L_ of <25 cmH_2_O was considered to be lung protective ventilation. PEEP was titrated on the basis of a positive end-expiratory P_L_ and electrical impedance tomography.
